# Towards non-invasive imaging through spinal-cord generated magnetic fields

**DOI:** 10.3389/fmedt.2024.1470970

**Published:** 2024-10-09

**Authors:** Meaghan E. Spedden, George C. O’Neill, Tim M. Tierney, Timothy O. West, Maike Schmidt, Stephanie Mellor, Simon F. Farmer, Sven Bestmann, Gareth R. Barnes

**Affiliations:** ^1^ Department of Imaging Neuroscience, Institute of Neurology, University College London, London, United Kingdom; ^2^ Department of Neuroscience, Physiology and Pharmacology, University College London, London, United Kingdom; ^3^ Department of Biomedical Engineering, Imperial College London, London, United Kingdom; ^4^ Department for Clinical and Movement Neuroscience, UCL Queen Square Institute of Neurology, University College London, London, United Kingdom; ^5^ Department of Clinical Neurology, The National Hospital for Neurology and Neurosurgery, London, United Kingdom

**Keywords:** optically pumped magnetometer (OPM), superconducting quantum interface devices (SQUIDs), human spinal cord, sensorimotor control, neuroimaging (functional)

## Abstract

Non-invasive imaging of the human spinal cord is a vital tool for understanding the mechanisms underlying its functions in both healthy and pathological conditions. However, non-invasive imaging presents a significant methodological challenge because the spinal cord is difficult to access with conventional neurophysiological approaches, due to its proximity to other organs and muscles, as well as the physiological movements caused by respiration, heartbeats, and cerebrospinal fluid (CSF) flow. Here, we discuss the present state and future directions of spinal cord imaging, with a focus on the estimation of current flow through magnetic field measurements. We discuss existing cryogenic (superconducting) and non-cryogenic (optically-pumped magnetometer-based, OPM) systems, and highlight their strengths and limitations for studying human spinal cord function. While significant challenges remain, particularly in source imaging and interference rejection, magnetic field-based neuroimaging offers a novel avenue for advancing research in various areas. These include sensorimotor processing, cortico-spinal interplay, brain and spinal cord plasticity during learning and recovery from injury, and pain perception. Additionally, this technology holds promise for diagnosing and optimizing the treatment of spinal cord disorders.

## 1 Introduction

The human spinal cord dynamically transmits, modulates, and integrates neural signals between the brain and the periphery to facilitate movement, somatosensory processing, and autonomic function (1–4). To understand the mechanisms underlying these functions in physiological and pathological conditions, being able to measure the spatial and temporal dynamics of spinal cord activity non-invasively in humans is paramount.

Spinal cord imaging in humans is notoriously difficult for several reasons (5, 6). The spinal cord resides within the spinal canal and is surrounded by the bony vertebrae, cartilaginous discs, the dura and cerebrospinal fluid (CSF) and at its widest point is only about 15 mm in diameter wide (7). The spinal cord is also situated posteriorly in the torso, with the epidural space being several centimetres away from the surface of the back (8). This anatomical configuration creates a highly challenging imaging environment. Physiological motion caused by respiration and the movement of the heart (9) add further complexity to the imaging process. Additionally, flow and pulsation of the CSF that surrounds the spinal cord can cause it to move, particularly in areas closer to the head region (10). Finally, recording activity from the brain and spinal cord simultaneously poses an even greater challenge (11). Nonetheless, interactions between the brain and spinal cord underpin the vast repertoire of human behaviour and autonomic function, and thus cortico-spinal imaging forms a major goal for the field.

The development of non-invasive imaging methods is crucial, particularly in clinical scenarios where invasive techniques could exacerbate existing conditions or pose significant risks to the patient. For example, in cases of spinal cord injury, non-invasive imaging allows for the monitoring of spinal cord functional integrity without introducing additional trauma. Similarly, in chronic conditions such as degenerative disc disease and spinal stenosis, repeated assessments are often necessary. Non-invasive methods provide a safe and effective means of tracking disease progression and treatment efficacy, reducing the potential for complications associated with more invasive procedures. Non-invasive imaging also extends applications to populations in which invasive procedures are not typically permitted, e.g., healthy neurotypical participants.

The challenges of spinal cord imaging have posed a limit to our understanding of the neurophysiology of the human spinal cord and emphasise the need for novel approaches that can enhance our basic understanding of healthy spinal cord function, enable better diagnosis and treatment of spinal cord injuries, and help predict clinical outcomes (3).

## 2 Minimally- and non-invasive recording techniques

While directly studying spinal cord physiology is clearly challenging, the continuing development of novel recording techniques provide evidence that neurophysiological assessment of the human spinal cord is feasible.

### 2.1 Minimally invasive techniques

Minimally invasive techniques, such as the implantation of flexible microelectrode arrays for spinal cord stimulation (SCS), allow for the recording of epidural signals, including evoked compound action potentials that quantify the spinal cord's response to electrical stimulation (12–14). Despite challenges in isolating spinal cord signals from myogenic responses and stimulation artefacts (15), these recordings offer valuable insights for optimizing SCS programming. Another emerging technique, functional ultrasound imaging (fUSI), has recently been applied to the human spinal cord (16), demonstrating its ability to capture evoked hemodynamic changes with superior spatiotemporal resolution compared to fMRI (17). Though currently minimally invasive, fUSI holds promise for fully non-invasive spinal cord imaging in the near future.

### 2.2 Functional magnetic resonance imaging of the human spinal cord

Functional magnetic resonance imaging (fMRI) of the human spinal cord was introduced in the mid 1990's by Yoshizawa and colleagues, who demonstrated changes in blood oxygen level dependent (BOLD) spinal activity during a simple motor task consisting of opening and closing the hand (18). Since then it has been used to validate functional sensorimotor pathways, outline pain networks in the spinal cord, map resting state networks, and study spinal cord pathologies (3, 19–23). The first demonstration of concurrent brain and spinal cord fMRI was also recently published (24), which opens up possibilities for studying the integrated functional organisation of sensorimotor networks in the entire central nervous system.

While it is clear that immense progress has been made by this modality, spinal cord fMRI is technically challenging (5, 25) and performed by relatively few research groups (5), which means that standards of acquisition and processing are not yet well established. Notwithstanding its unrivalled spatial precision, fMRI relies on neurovascular coupling as an indirect readout of neural activity, with a temporal resolution on the scale of seconds ill-suited for the study of fast changes in neural activity.

### 2.3 Electrospinography

There is a significant body of literature on non-invasive electrospinography (ESG) for recording somatosensory evoked potentials (SEP) (26–30). This work typically uses surface electrodes positioned on the neck or back to capture spinal cord SEPs using time-locked averaging over a high number of repeats of peripheral nerve stimulation. ESG can be used in the clinic to record canonical evoked potentials originating from the spinal cord, e.g., to test the integrity of pathways in patients with myelopathy (31). There has also been a recent resurgence of ESG, using novel high density electrode montages, aimed at studying basic spinal cord physiology (32–34). The advantages of ESG include its flexibility in electrode array configuration and good temporal resolution on the scale of milliseconds. Critically, it also provides a direct measure of neural ensemble activity. The spatial resolution of ESG is limited due to the ill-posed inverse problem and the resulting complexity of localizing the source of electrical activity from the recorded signals (35). This means that it is challenging to use this method to address questions about where in the spinal cord the signals measured by the electrodes originate.

### 2.4 Cryogenic magnetospinography: imaging of spinal cord activity

Similar to ESG, magnetic field-based imaging also provides a direct measure of neural activity and excellent temporal resolution on the order of milliseconds (36). It is also possible to obtain relatively good spatial information using magnetic fields because they are less distorted by the conductivities of the tissues surrounding the spinal cord than electrical fields (36, 37). This makes it more feasible to infer current sources from fields measured from the surface of the body (38).

Capitalising on these features, cryogenic magnetospinography (MSG) is a non-invasive method that records magnetic fields generated by spinal cord activity traditionally using super-conducting quantum interference devices (SQUIDs) to detect the weak magnetic fields that arise from neural activity (39–43). The operating principles of this system are detailed in a paper by Adachi and Kawabata in this special issue (43).

In the early 1990s, Curio and colleagues conducted the first human MSG recordings using a single SQUID magnetometer to record evoked fields from median nerve stimulation at the wrist (39). Building on this work, they soon demonstrated the first mappings of evoked fields from the lumbar spinal cord in both healthy individuals (40) and patients with nerve root compression (44). This group also pioneered recordings of magnetic fields from peripheral nerves (45).

**Figure 1:**
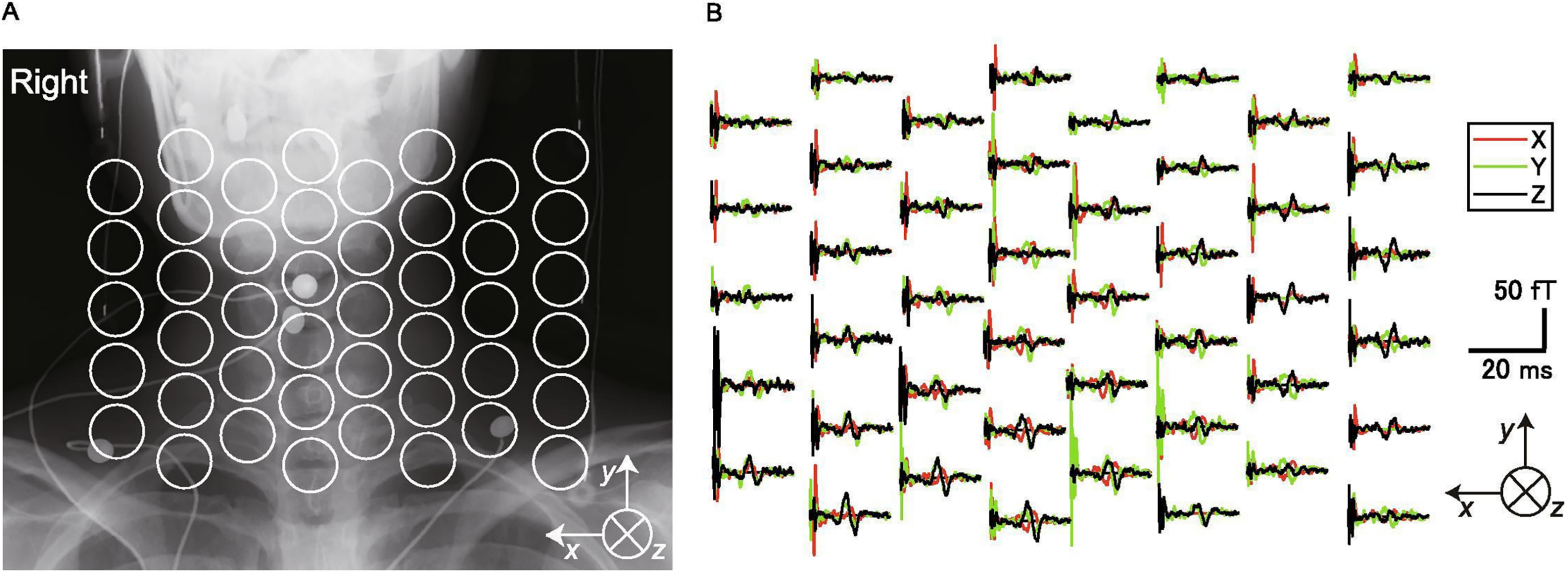
Cryogenic magentospinography (MSG). **(A)** Positions of the SQUID sensors superimposed on an x-ray image of a participant. **(B)** The magnetic fields recorded by each sensor (single participant). The black traces are magnetic fields in the ventral–dorsal direction relative to the cervical spinal cord (ventral is upward in the graphs). The red traces are magnetic fields in the left–right direction (right is upward). The green traces are magnetic fields parallel to the spinal cord (cranial is upward). This figure is reproduced from Akaza et al. (38) “Noninvasive measurement of sensory action currents in the cervical cord by magnetospinography”, Clinical Neurophysiology, 2021, licensed under CC BY-NC-ND 4.0 https://creativecommons.org/licenses/by-nc-nd/4.0/.

Since the late 1990's one research group has pursued this approach to develop a commercially available MSG system (Kanazawa Institute of Technology, Nonoichi, Japan; RICOH Co., Ltd., Tokyo, Japan) (43, 46) which uses a SQUID-based system specifically tailored for recording spinal cord evoked fields (42, 46–48) (Figure 1). Currently, this MSG system consists of a cryostat with a main body holding liquid helium and a fixed sensor array of 44 vector gradiometers in a 188 × 150 mm area installed in a protrusion on which the participant can lie supine (46). Measurements are performed in a magnetically-shielded room (MSR) where x-ray images are also acquired *in situ*, allowing functional activity to be superimposed on structural images.

Initial studies with this approach quantified the magnetic fields originating from the cervical spinal cord following stimulation of the lower thoracic cord in rabbits and cats, demonstrating that MSG can capture the transmission of ascending volleys of action potentials along the spinal cord (49, 50). This work further showed that it was possible to detect conduction blocks due to experimentally induced lesions. Animal models were also used to demonstrate that MSG could detect two types of neural activity: conduction action potentials and stationary synaptic activity (51).

In humans, MSG has been employed to record cervical spinal cord evoked fields after spinal cord stimulation with epidural electrodes and to identify the point of cervical spinal stenosis in a patient with cervical spondylotic myelopathy (41). In a series of studies, it has been demonstrated that MSG is capable of robustly detecting evoked fields in the cervical (38, 41), thoracic (52), and lumbar (53, 54) spinal cord in response to peripheral nerve stimulation. These evoked fields also allow for reconstruction of current flow in the spinal cord, and the resulting patterns of activity are in agreement with the known physiology of sensory pathways (38).

Together, this compelling body of work highlights the ability of cryogenic magnetic field-based imaging to reliably record evoked spinal cord activity in humans, with potential for the diagnosis of functional disturbances of spinal cord function such as spinal cord myelopathy.

### 2.5 Magneto-spino-encephalography: concurrent imaging of brain and spinal cord activity with optically-pumped magnetometers

The MSG work by Kawabata and colleagues (43) has demonstrated the feasibility of measuring the weak magnetic fields generated by spinal cord activity non-invasively. However, a significant challenge with SQUID-based systems is their need for cryogenic cooling, making them heavy, stationary, and expensive to redesign. Importantly, the subject must also remain still with their spinal cord positioned close to the sensor array. Furthermore, the existing sensor array also has a relatively limited field of view, which entails that covering larger regions of the spinal cord requires many repetitions of an experiment with the participant in different positions (55).

Conversely, a newer generation of magnetic sensors, optically-pumped magnetometers (OPMs), do not require cryogenic cooling (56–58). Over the last 10 years, OPMs have been used to develop a new approach to magnetoencephalography (MEG) (57–59), and the technique shows significant potential for spinal cord imaging. OPMs leverage the quantum properties of atoms to detect local magnetic fields, with various sensors developed using different atomic vapors. For a detailed explanation of the operating principles of these sensors, please refer to references (57) (alkali-based OPMs) and (60) (helium-based OPMs).

OPM-based imaging offers three key advantages over cryogenic systems:

1. It permits multiple simultaneous recordings from different parts of the body, such as the spinal cord, muscle, or nerve (56, 61–63), and the brain.
2. It allows construction of sensor arrays specific to an experimental question or individual's body shape (56, 64, 65).
3. Theoretically, the whole neuro-axis can be studied during natural movement (66, 67). This has significant implications for both neuroscientific and clinical paradigms that involve large scale movement, such as walking for example. Note however that this presents a significant challenge to managing interference (see Section 3).

**Figure 2:**
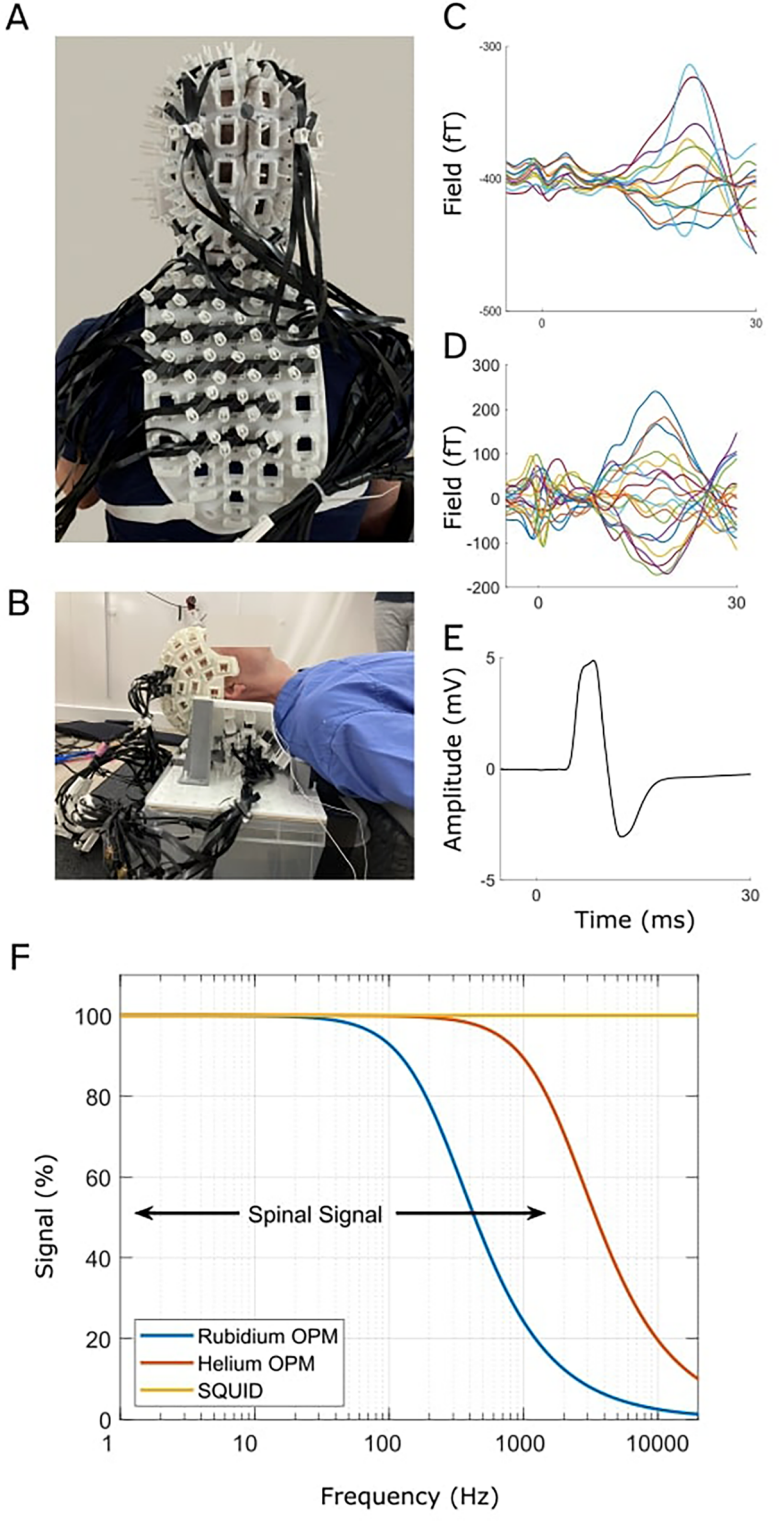
OPM-based magneto-spino-encephalography (OP-MSEG). This system comprises custom-built 3D printed rigid scanner casts that position OPMs on the head, neck, and back to record from the brain and spinal cord concurrently. A and B show two recent scanner casts constructed to record from participants in the seated position **(A)** or lying down **(B)**. Evoked fields in the cortex **(C)** and spinal cord **(D)**, recorded with OPMs, and evoked potential from abductor pollicis brevis (APB) (thumb) muscle **(E)** recorded with EMG, in response to median nerve stimulation at the wrist. Data are from a single participant. **(F)** Comparison of OPM and SQUID sensor bandwidth. Bandwidths are illustrated as signal strength (magnitude) as a function of frequency (logarithmic axis). Black arrows show the approximate bandwidth of spinal cord activity. While SQUID sensors have the largest bandwidth, OPMs are able to capture a significant component of the spinal cord signal. **(A**–**E)** are adapted from Mardell et al. (56), “Concurrent spinal and brain imaging with optically pumped magnetometers,” Journal of Neuroscience Methods, 2024, licensed under CC BY 4.0: https://creativecommons.org/licenses/by/4.0/.

There is however one main disadvantage of OPMs over SQUID systems. The bandwidth (up to 40 kHz) of SQUID systems allows them to record signals separated in time by fractions of milliseconds. For example, it is possible to directly observe the propagation of intra-axonal current flow along the cord or peripheral nerves at conduction velocities of 60–100 m/s (43). In contrast, the bandwidth of the OPM system is determined by the properties of the vapour being optically pumped (Figure 2F). Typically, there is a trade-off in bandwidth against sensitivity, and current commercial OPMs range in bandwidth from 150 Hz to around 2 kHz. That said, OPM technology continues to progress, and recent work (62) has demonstrated the potential of OPMs in peripheral nerve neurography and their ability to characterise action potential currents. Also, we know from invasive recordings (51), and recent empirical work (56, 61) that we expect low frequency fields from the spinal cord, possibly due to post-synaptic current flow.

It is worth noting that OPM measurements can be impeded by cross-axis projection errors (CAPE), where the presence of large background magnetic fields can introduce gain and orientation errors across the frequency spectrum in the OPM output signal (68). Thankfully, this issue can be addressed using closed loop modes of operation. In this case, internal coils continuously null the field at each sensor, preventing the gain and orientation errors. All OPM manufacturers provide some implementation of closed loop operation but with varying bandwidth and dynamic range (69–71). Currently, Helium based OPMs exhibit particular promise because they feature a very large bandwidth (2,000 Hz) and high dynamic range (∼250 nT). They cover the full range of signals observable from the spinal cord while effectively mitigating the nonlinearities that can be encountered when working with OPMs due to CAPE.

Recently, our research group has begun developing a system using OPMs to record both brain and spinal cord activity concurrently, termed magneto-spino-encephalography (OP-MSEG) (56, 61). There is also the potential to simultaneously record muscle activity (EMG) and kinematics (limb acceleration). The OP-MSEG system utilizes custom-built, 3D printed scanner casts that house OPMs in custom arrays for the head and back (Figures 2A,B). We have printed custom scanner casts constructed from optical scans of participant neck and back shape (56), as well as generic casts (61), and have adapted cast designs to permit recordings in various positions, including both lying down and seated upright (56, 61). Note however that we currently record from participants in the supine position to minimize the influence of neck and back muscle activity on spinal cord signals as we are still working towards optimizing interference rejection to fully separate the two signal sources (56) (see Section 3).

We currently utilize Quspin manufactured triaxial OPMs (Louisville, CO) which have a sensitivity of approximately 15 fT/√Hz in the 10–100 Hz range, but they can detect activity up to 500 Hz, albeit with reduced sensitivity (62). For example, the frequency response of our sensors predicts a reduction in signal strength by approx. 2 at 500 Hz. However, this is offset by the increased proximity to the spinal cord (relative to cryogenic sensors), resulting in a minimal net signal loss. Note that recently introduced OPM sensors bypass this signal loss completely as they have bandwidths up to 2,000 Hz and are able to take full advantage of the increased signal due to proximity (72). Our OPM recordings are performed in an MSR (Magnetic Shields, Ltd, Staplehurst, UK) with two inner layers of 1 mm mu-metal, a 6 mm copper layer and two external layers of 1.5 mm mu-metal.

In our initial proof-of-principle work (56), we recorded spinal and cortical responses to median nerve stimulation at the wrist (Figures 2C–E). The evoked spinal and cortical magnetic fields exhibited the expected latencies, i.e., consistent with conduction times, and showed good correspondence with SEPs, their electrical analogue. We also found evidence for longer latency spinal cord responses that are less well described in existing literature, but are thought to either reflect intra-spinal mechanisms, descending feedback signals, or long-latency reflex activity (33, 73). This work is significant because it demonstrates the novel capability for concurrent, non-invasive, millisecond imaging of both brain and spinal cord activity using OPMs.

Extending this research, we are currently leveraging our capability to simultaneously record brain and spinal cord activity to study their natural interactions during voluntary movement. In these experiments (61) we recorded brain and spinal cord activity during a simple hand contraction, along with electromyography (EMG) from the active muscle, to quantify functional connectivity between the three regions. This preliminary work found evidence for significant rhythmic interactions between the spinal cord and brain and muscle activity in the 5–35 Hz frequency range, suggesting it is feasible to use OP-MSEG to study functional integration between the CNS and periphery during simple movement. We posit that these low frequency endogenous rhythms derive from post-synaptic rather than axonal current flow.

Collectively, this initial research suggests that OPMs could represent a major advancement in spinal cord imaging by providing flexible access to both the spinal cord and brain, and facilitating the study of naturalistic movement. However, this method is still in its early stages and faces several notable challenges.

## 3 Challenges for measuring spinal cord activity with magnetic fields

Using magnetic fields to record spinal cord activity offers promising opportunities, but this approach faces significant challenges that must be addressed to advance the field. Here, we briefly highlight challenges in two areas: interference rejection and source modelling (estimating spinal cord current flow).

### 3.1 Interference rejection

Isolating spinal cord signals from environmental noise, motion-related field changes, and muscle activity is challenging with OPMs as magnetometers have no inherent interference rejection capabilities. Cryogenic MEG and MSG systems have successfully tackled this using triaxial gradiometer configurations (41, 74) and analytical methods like Dual Signal Subspace Projection (DSSP) (75). Multi-axis field measurements are extremely useful for distinguishing signals from interference in both the brain (76–78) and spinal cord (41). However, it remains untested how effectively magnetometers can achieve this separation for spinal cord recordings (79). Analytical methods, such as DSSP, rely on prior knowledge of participant anatomy to separate signals from different regions, but these assume no relative movement between the spinal cord and sensors. We are optimistic of the capacity for OPM studies of the spinal cord to capture natural human movement (80). Achieving this will require accounting for the natural movement of the spinal cord relative to the sensors. These challenges can likely be addressed in part with comprehensive simulations. However, these require a robust forward model describing how spinal cord neural activity translates to magnetic fields detected by OPMs.

### 3.2 Current flow estimates in the spinal cord

The aim of source modelling is to estimate the current flow in the spinal cord that gives rise to the observed changes in magnetic field. Source modelling of brain activity has been developed extensively for over 30 years (81–85), and some of these tools are likely also useful for the spinal cord. To date there have been some preliminary demonstrations of source localising MSG data in fixed systems (41, 56, 61). However, if we are to develop a fully wearable and mobile system for imaging the entirety of the human nervous system there are key technical challenges that need to be addressed: (i) accurately locating the anatomy of the spinal cord relative to the sensors, (ii) optimizing sensor array configurations, and (iii) developing reliable volume conduction models. First, the mobility of the vertebral column and spinal cord (e.g., relative to the skull and brain) entails that small postural changes between anatomical and functional data registration can lead to unreliable estimates of spinal cord location and confound source analysis. Second, sensor arrays must be strategically configured to capture spinal cord activity at different depths, i.e., cervical vs. lumbar. We also need to know how to spatially sample back muscle activity if we want to adequately model (and remove it) from our data. Finally, the question of how to approximate the complexity of the spinal cord and torso for a solvable volume conduction model needs investigation. While complex numerical models may be needed due to the various tissue types between the spinal cord and body surface, the property of magnetic fields to propagate through different tissues with minimal distortion suggests that simpler models might be sufficient.

## 4 Future directions

In the clinic, cryogenic MSG (42) holds significant potential for localizing the region of nerve damage in the spinal cord. The technology is currently in the research phase, and we anticipate that its introduction into clinical settings will play a pivotal role in functional assessments of spinal cord disorders. For example, spondylotic myelopathy, a common condition in older adults caused by spinal canal narrowing, has an unpredictable progression and variable severity, even with similar levels of structural spinal cord compression on anatomical MRI (86–88). Using MSG for functional assessment of spinal cord activity will likely prove extremely useful for identifying conduction blocks, predicting disease progression, and formulating treatment strategies (41, 50, 89).

One of the attractions of OP-MSEG is its capacity for quantifying interactions between the brain, spinal cord, and periphery in a flexible way because of the adaptability of the sensor arrays. The ability to record activity non-invasively from the entire CNS, simultaneously and with good spatial and temporal resolution, provides a novel route for studying the control of human movement (90, 91), brain and spinal cord plasticity (92), and pain processing (93, 94), and how this functional circuity is affected by neurological disorders (95, 96). In the context of movement control and movement disorders, combining OP-MSEG with high-density surface EMG (HD-EMG) and kinematics could be highly beneficial. HD-EMG permits decomposition of the integrated EMG signal into single motor unit potentials (97), which in combination with OP-MSEG may give us unprecedented access to the different sources of activity within spinal cord circuits, and how these interact with the brain. For example, it may allow us to study the direct correlates of motor unit recruitment on the spinal cord level (98, 99).

Finally, OP-MSEG offers potential for studying the brain and spinal cord concurrently and non-invasively during natural movement. Although this introduces new challenges, it can provide access to manifold neuroscientific and clinical questions allowing the study of new patient groups (e.g., those who cannot comply with a supine stationary scanner) in new experimental or clinical paradigms. It also promises to advance research into the neural control of natural movement and allow us to study behaviour in increasingly ecologically valid settings.

## 5 Conclusion

Non-invasive imaging of the spinal cord presents numerous methodological challenges yet could give rise to significant scientific and clinical advances in our understanding of human physiology. Cryogenic MSG has proven robust for recording evoked fields from the spinal cord and will be of clinical relevance in detecting functionally significant spinal cord damage. Building on this foundation, OP-MSEG can image the brain and spinal cord simultaneously and permits participant movement. Although further development is needed, particularly in interference rejection and source imaging, OP-MSEG promises a range of important future applications. These include basic research into how the brain and spinal cord interact to control natural movement, as well as clinical investigations into chronic pain and motor disorders in humans.

## Data Availability

The original contributions presented in the study are included in the article/Supplementary Material, further inquiries can be directed to the corresponding author.
